# Configuring Balanced Scorecards for Measuring Health System Performance: Evidence from 5 Years' Evaluation in Afghanistan

**DOI:** 10.1371/journal.pmed.1001066

**Published:** 2011-07-26

**Authors:** Anbrasi Edward, Binay Kumar, Faizullah Kakar, Ahmad Shah Salehi, Gilbert Burnham, David H. Peters

**Affiliations:** 1 Department of International Health, Johns Hopkins Bloomberg School of Public Health, Baltimore, Maryland, United States of America; 2 Indian Institute of Public Health, Public Health Foundation of India, New Delhi, India; 3 Office of the President of Afghanistan, Kabul, Afghanistan; 4 Ministry of Public Health, Kabul, Afghanistan; London School of Hygiene and Tropical Medicine, United Kingdom

## Abstract

**Background:**

In 2004, Afghanistan pioneered a balanced scorecard (BSC) performance system to manage the delivery of primary health care services. This study examines the trends of 29 key performance indicators over a 5-year period between 2004 and 2008.

**Methods and Findings:**

Independent evaluations of performance in six domains were conducted annually through 5,500 patient observations and exit interviews and 1,500 provider interviews in >600 facilities selected by stratified random sampling in each province. Generalized estimating equation (GEE) models were used to assess trends in BSC parameters. There was a progressive improvement in the national median scores scaled from 0–100 between 2004 and 2008 in all six domains: patient and community satisfaction of services (65.3–84.5, *p<*0.0001); provider satisfaction (65.4–79.2, *p<*0.01); capacity for service provision (47.4–76.4, *p<*0.0001); quality of services (40.5–67.4, *p<*0.0001); and overall vision for pro-poor and pro-female health services (52.0–52.6). The financial domain also showed improvement until 2007 (84.4–95.7, *p<*0.01), after which user fees were eliminated. By 2008, all provinces achieved the upper benchmark of national median set in 2004.

**Conclusions:**

The BSC has been successfully employed to assess and improve health service capacity and service delivery using performance benchmarking during the 5-year period. However, scorecard reconfigurations are needed to integrate effectiveness and efficiency measures and accommodate changes in health systems policy and strategy architecture to ensure its continued relevance and effectiveness as a comprehensive health system performance measure. The process of BSC design and implementation can serve as a valuable prototype for health policy planners managing performance in similar health care contexts.

*Please see later in the article for the Editors' Summary*

## Abstract 2

Anbrasi Edward and colleagues report the results of a balanced scorecard performance system used to examine 29 key performance indicators over a 5-year period in Afghanistan, between 2004 and 2008.

## Editors' Summary

**Background:**

Traditionally, the performance of a health system (the complete network of health care agencies, facilities, and providers in a defined geographical region) has been measured in terms of health outcomes: how many people have been treated, how many got better, and how many died. But, nowadays, with increased demand for improved governance and accountability, policy makers are seeking comprehensive performance measures that show in detail how innovations designed to strengthen health systems are affecting service delivery and health outcomes. One such performance measure is the “balanced scorecard,” an integrated management and measurement tool that enables organizations to clarify their vision and strategy and translate them into action. The balanced scorecard—essentially a list of key performance indicators and performance benchmarks in several domains—was originally developed for industry but is now becoming a popular strategic management tool in the health sector. For example, balanced scorecards have been successfully integrated into the Dutch and Italian public health care systems.

**Why Was This Study Done?:**

Little is known about the use of balanced scorecards in the national public health care systems of developing countries but the introduction of performance management into health system reform in fragile states in particular (developing countries where the state fails to perform the fundamental functions necessary to meet its citizens' basic needs and expectations) could help to promote governance and leadership, and facilitate essential policy changes. One fragile state that has introduced the balanced scorecard system for public health care management is Afghanistan, which emerged from decades of conflict in 2002 with some of the world's worst health indicators. To deal with an extremely high burden of disease, the Ministry of Public Health (MOPH) designed a Basic Package of Health Services (BPHS), which is delivered by nongovernmental organizations and MOPH agencies. In 2004, the MOPH introduced the National Health Service Performance Assessment (NHSPA), an annual country-wide assessment of service provision and patient satisfaction and pioneered a balanced scorecard, which uses data collected in the NHSPA, to manage the delivery of primary health care services. In this study, the researchers examine the trends between 2004 and 2008 of the 29 key performance indicators in six domains included in this balanced scorecard, and consider the potential and limitations of the scorecard as a management tool to measure and improve health service delivery in Afghanistan and other similar countries.

**What Did the Researchers Do and Find?:**

Each year of the study, a random sample of 25 facilities (district hospitals and comprehensive and basic health centers) in 28 of Afghanistan's 34 provinces was chosen (one province did not have functional facilities in 2004 and the other five missing provinces were inaccessible because of ongoing conflicts). NHSPA surveyors collected approximately 5,000 patient observations, 5,000 exit interviews with patients or their caregivers, and 1,500 health provider interviews by observing consultations involving five children under 5 years old and five patients over 5 years old in each facility. The researchers then used this information to evaluate the key performance indicators in the balanced scorecard and a statistical method called generalized estimating equation modeling to assess trends in these indicators. They report that there was a progressive improvement in national average scores in all six domains (patients and community satisfaction with services, provider satisfaction, capacity for service provision, quality of services, overall vision for pro-poor and pro-female health services, and financial systems) between 2004 and 2008.

**What Do These Findings Mean?:**

These findings suggest that the balanced scorecard was successfully used to improve health system capacity and service delivery through performance benchmarking over the 5-year study period. Importantly, the use of the balanced scorecard helped to show the effects of investments, facilitate policy change, and create a more evidence-based decision-making culture in Afghanistan's primary health care system. However, the researchers warn that the continuing success of the balanced scorecard in Afghanistan will depend on its ability to accommodate changes in health systems policy. Furthermore, reconfigurations of the scorecard are needed to include measures of the overall effectiveness and efficiency of the health system such as mortality rates. More generally, the researchers conclude that the balanced scorecard offers a promising measure of health system performance that could be used to examine the effectiveness of health care strategies and innovations in other fragile and developing countries.

**Additional Information:**

Please access these Web sites via the online version of this summary at http://dx.doi.org/10.1371/journal.pmed.1001066.

- A 2010 article entitled An Afghan Success Story: The Balanced Scorecard and Improved Health Services in *The Globe*, a newsletter produced by the Department of International Health at the John Hopkins Bloomberg School of Public Health, provides a detailed description of the balanced scorecard used in this study
- Wikipedia has a page on health systems and on balanced scorecards (note that Wikipedia is a free online encyclopedia that anyone can edit; available in several languages)
- The World Health Organization country profile of Afghanistan provides information on the country's health system and burden of disease (in several languages)

## Introduction

Emerging from decades of conflict, in 2002, Afghanistan had some of the worst health indicators in the world, with more than 30% of its population living below the poverty line. A myriad of environmental and security constraints further exacerbate the deficits in the health system, impeding optimal service delivery. Efforts to reconstruct Afghanistan's heath infrastructure have accelerated since, with the total public health spending rising to US$280 million in 2008–2009, with most (85%) financed by external donor assistance [1]. To address the exceptionally high disease burden, the Under Five Mortality Rate estimated to be 257 deaths per 1,000 live births and the Maternal Mortality Ratio estimated at 1,600 deaths per 100,000 births [2]–[5], the Ministry of Public Health (MOPH) designed a Basic Package of Health Services (BPHS) delivered primarily through contracting mechanisms with nongovernmental organization (NGO) and MOPH implementing agencies [6]. To measure the performance of the BPHS, in 2004, the MOPH initiated the National Health Service Performance Assessments (NHSPA), which has been conducted annually in every accessible province.

In the current era where there is increasing demand for improved governance and accountability, policy makers seek comprehensive performance measures that illustrate evidence of health systems strengthening innovations on service delivery and health outcomes [7]. Among the conceptual frameworks employed for measuring organizational performance, the balanced scorecard (BSC), developed for industry by Kaplan and Norton, has steadily gained momentum as a popular strategic management tool in the health sector [8]–[12]. In comparison to the traditional performance metrics that measure health outcomes, the scorecard offers an integrated measurement and management system, that links the mission and policy of the organization through strategic mapping of multiple performance domains facilitating benchmarking and fostering a culture of accountability [11],[13].

Successes of scorecard integration have been reported in the Dutch and Italian public health care systems, but aside from two recent publications in a hospital in Pakistan and a district level program in temporary health clinics to accommodate displaced populations, their application in national public health systems in low-income countries is limited [14]–[17]. In 2004, Afghanistan pioneered a BSC integrating the health sector's vision and strategy to measure performance of service capacity and delivery on the basis of the standards instituted in the BPHS, which prioritizes services to address the major disease burden in vulnerable population segments (Box 1). The traditional BSC quadrants were modified to include six domains with 29 core performance indicators, designed by a multidisciplinary team of government, donor, and NGO stakeholders. The “Patient and Community Perspectives domain” focused on patient satisfaction and the engagement of community councils (*shura-e-sehies)*. Workforce capacity, management, and satisfaction was measured by the “Staff domain” followed by a set of indicators examining system preparedness based on BPHS standards for staffing, equipment, essential commodities, and infrastructure in the “Capacity for Service Provision domain.” Service provision and clinical quality of care was measured through the “Service Provision domain.” The “Financial Systems domain” included aspects regarding user fees. This domain was eliminated in 2008, following the discontinuation of user fees after 2007. The last domain on “Overall Vision” measured equity factors. Earlier publications illustrated the process undertaken for scorecard design and some evidence of management decision-making using scorecards results [13],[18]. The purpose of this study is to illustrate the performance trends in delivering the BPHS during the first 5-y period following elections in 2004, and to reflect on the potential and limitations of the scorecard as a performance management tool to measure and improve health service delivery in similar health care contexts.

**Box 1. Seven Elements of BPHS**

1. Maternal and newborn health (ANC, PNC, delivery, family planning, care of the new born)
2. Child health and immunization (EPI and IMCI)
3. Public nutrition (prevention, assessment and treatment of malnutrition)
4. Communicable disease treatment and control (TB, malaria, and HIV/AIDS)
5. Mental health (education, awareness, case detection, identification and treatment of mental illness)
6. Disability services (awareness, prevention, education, assessment and referral)
7. Regular supply of essential drugs

ANC, antenatal care ; EPI, expanded program for immunization; IMCI, integrated management of childhood illness; PNC, postnatal care; TB, tuberculosis.

## Methods

The BSC was designed to integrate key performance indicators from the NHSPA conducted annually between 2004 and 2008. Employing a multistage stratified random sampling every year, a sample of 25 facilities were selected from each province, according to the following distribution by facility type: three district hospitals; seven comprehensive health centers; 15 basic health centers. However, the sampling frame varied each year as (a) new facilities were being constructed; (b) some provinces had fewer functional facilities than the proposed sample and therefore all functional facilities were included; and (c) adverse security incidents prevented surveyors from accessing sampled health facilities in some provinces. Therefore up to 25 facilities were included in each province. Weighting was applied at the provincial level on the basis of the total number of health facilities included at the national level, and subsequently national scores were computed on the basis of the weighted scores. The total national sample included in the scorecard represented approximately 50% of the functional health facilities in Afghanistan. The inclusion of subcenters and mobile clinics, subsequently, has necessitated the sampling of these facilities in the national assessments in 2009. However these peripheral facilities were not included in the national performance assessments between 2004 and 2008.

In this study, we included data from all 28 provinces that were surveyed annually between 2004 and 2008. Six of the 34 provinces in Afghanistan were not included in the trend analysis, as Daikundi province did not have functional facilities in 2004, and Helmand, Kandahar, Zabul, and Uruzgan were excluded after 2004 and subsequently Farah, in 2008 as they were inaccessible due to worsening security.

A five-member survey team conducted the assessments in the selected facilities in each province. In each facility, observations of patient consultations were conducted on five children under 5 y, and five patients above 5 y, selected by systematic random sampling using a sampling interval on the basis of utilization rates, resulting in an annual national sample of approximately 5,000 patient observations, 5,000 exit interviews with patients or caretakers of children under 5 y, and 1,500 health provider interviews. The assessment of provider knowledge was modified in 2008, with the introduction of case vignettes to determine the provider's ability to diagnose and treat conditions on the basis of presenting symptoms. Response rate was >99% for providers and patients interviewed.

Each of the 29 BSC indicators was converted to a percentage score ranging from 0 to 100. An unadjusted mean score for each province and indicator was first calculated by averaging the performance of the sampled facilities in that province and weighted to account for the different proportion of facility types in the total national sample. A national score for each indicator was calculated as a median of weighted provincial mean scores. Provincial performance in 2004 was applied to set the benchmarks and provinces were categorized into quintile groups on the basis of performance with the top and bottom quintiles illustrating upper and lower benchmarks. Fourteen of the 29 indicators were indices, created from an aggregate set of performance indicators.

Wilcoxon matched-pair signed-rank test was applied to test the difference in the scores between 2004 and 2008, using STATA 10 (Stata Corp). We used generalized estimating equation (GEE) with robust standard errors using time as the predictor to assess the linear changes for each indicator over the 5-y period, which accounts for the correlation between repeated observations at the provincial level [19].

The study was considered exempt research according to the Johns Hopkins University Policy for Human Subjects Research and had approval from the MOPH in Afghanistan. A detailed description of the sampling methodology, survey procedures, scoring system, costs, and data quality measures undertaken in the NHSPA have been reported previously [13],[18]. This paper analyzes performance trends in BPHS service delivery from the 28 provinces (of 34) that were included in all years between 2004 and 2008 (Table 1).

**Table 1:** Sample of provinces, health facilities, health providers, and patients.

Sampling Profile	Year of Assessment				
	2004	2005	2006	2007	2008
Provinces	28	28	28	28	28
Facilities	551	612	612	615	600
Patient observations: under 5 y	2,504	2,758	2,892	2,887	2,875
Patient observations: above 5 y	2,630	2,947	2,965	3,008	2,921
Total patient observations	5,134	5,705	5,857	5,895	5,796
Caretaker exit interviews of children under 5 y	2,458	2,755	2,892	2,887	2,875
Patient exit interviews: above 5 y	2,595	2,961	2,965	3,008	2,921
Total exit interviews	5,053	5,716	5,857	5,895	5,796
Health provider interviews	1,388	1,418	1,666	1,827	2,175

## Results

The national median performance score for the indicators included in each domain of the scorecard between 2004 and 2008 is illustrated in Table 2. Twenty of these indicators showed significant improvement (*p<*0.05) since 2004. Domains of service capacity and delivery, which scored poorly (<50 median score) in 2004, had more dramatic improvements in performance over the years than the domain for patient and community satisfaction that scored high initially (>65).

**Table 2:** National score by performance domain and indicator for 28 provinces from 2004 to 2008.

BSC Domains	National Median						
	Year of Assessment					Percent Change	
	2004	2005	2006	2007	2008	2004–2007	2004–2008
*Domain A: patients and community*	*65.3*	*70.8*	*80.0*	*81.4*	*84.5*	*+16.1* ***	*+19.2* ***
Overall patient satisfaction	84.7	86.9	86.4	77.7	81.0	−7.0	−3.7
Patient perception of quality index^a^	75.7	76.2	80.4	77.6	79.4	+1.9	+3.7*
Written *shura-e-sehie* activities	34.3	57.2	67.5	89.7	94.6	+55.3***	+60.2***
*Domain B: staff*	*65.4*	*76.3*	*74.3*	*79.6*	*79.2*	*+14.1* *	*+13.7* *
Health worker satisfaction index^a^	63.5	64.1	68.2	69.0	69.7	+5.5***	+6.2***
Salary payments current	66.2	88.7	80.5	90.7	86.0	+24.5*	+19.8*
*Domain C: capacity for service provision*	*47.4*	*57.5*	*66.3*	*73.0*	*76.4*	*+25.5* ***	*+29.0* ***
Equipment functionality index^a^	65.6	67.0	78.7	83.8	88.4	+18.2***	+22.9***
Drug availability index^a^	69.5	82.9	82.8	81.0	86.5	+11.5***	+17.0***
Family planning availability index^a^	62.1	64.7	84.9	93.7	95.1	+31.6***	+33.0***
Laboratory functionality index (hospitals and CHCs)^a^	19.1	36.3	43.3	58.5	64.9	+39.4***	+45.8***
Staffing index^a^	36.5	58.0	65.9	61.4	70.7	+25.0***	+34.2***
Provider knowledge score	54.7	69.0	69.3	68.7	79.7	+14.0***	NA
Training in the past year	39.0	74.3	68.4	68.5	70.7	+29.5***	+31.7***
HMIS use index^a^	68.5	65.9	78.4	92.6	92.6	+24.1***	+24.1***
Clinical guidelines index^a^	34.6	47.7	64.1	78.3	84.4	+43.7***	+49.8***
Infrastructure index^a^	54.5	44.3	48.7	54.6	55.2	+0.1	+0.6
Patient record index^a^	66.2	63.2	69.4	70.0	70.1	+3.8	+3.9
Facilities with TB registers	15.9	22.9	38.5	58.1	64.4	+42.1***	+48.4***
*Domain D: service provision*	*40.5*	*46.4*	*54.3*	*63.9*	*67.4*	*+23.4* ***	*+26.9* ***
Patient history and physical exam index^†^	71.1	73.5	82.9	83.4	84.7	+12.2***	+13.6**
Patient counseling index^a^	34.1	36.0	37.0	50.6	48.0	+16.5*	+14.0*
Proper sharps disposal	58.3	50.6	77.5	86.7	75.1	+28.4*	+16.8*
Average new outpatient visit per month (BHC>750 visits)	22.2	35.6	58.1	62.0	85.0	+39.8*	+62.7***
Patient consultation time (≥ 10 min)	19.8	6.2	7.0	17.0	20.3	−2.8	+0.6
BPHS facilities providing ANC	57.1	78.3	84.7	93.3	95.5	+36.2***	+38.4***
Delivery care according to BPHS	24.9	22.3	42.3	58.3	70.9	+33.4***	+46.0***
*Domain E: financial systems*	*84.4*	*85.3*	*89.8*	*95.7*	*NA*	*11.3* *	*NA*
Facilities with user fee guidelines	89.5	86.5	83.4	96.0	66.5	+6.5	NA
Facilities with exemptions for poor patients	82.5	91.7	100	100	100	+17.5***	NA
*Domain F: overall vision*	*52.0*	*52.9*	*52.8*	*53.5*	*52.6*	*+1.6* *	*+0.6* *
Females as percent of new outpatients	55.2	57.3	58.9	60	60.1	+4.8**	+4.9***
Outpatient visit concentration index^a^	50.2	50.7	51.2	50	50	−0.2	−0.2
Patient satisfaction concentration index^a^	50.0	49.8	49.8	49.6	49.6	−0.4	−0.4

a Aggregate of individual indicators.

*p<0.05.

**p<0.001.

***p<0.0001.

CHC,comprehensive health center.

### Patient and Community Domain

Overall, the patient and community domain illustrated significant improvement from baseline to 2008 (65.3%–84.5% *p<*0.0001). The substantial increases in this domain were primarily driven by the improvements in the engagement of village councils (*shura-e-sehie)* in the management oversight of health facilities measured by meeting records at the health facilities (<35% in 2004 to 95% in 2008). Client satisfaction and perception of quality, critical determinants to service utilization, were rated high (>70%) in all survey years. The annual increase for patient perception of quality and village council activities, estimated by GEE was significant (*p* = 0.02 and *p<*0.0001) (Table 3), although the size of the actual increases in patient-perceived quality was minimal, over the years.

**Table 3:** BSC indicators with statistically significant increments in mean scores between 2004 and 2008 using GEE robust estimates.

BSc Indicators	Annual Mean Score Increments		
	Coefficient	Confidence Interval	Standard Error
Patient perception of quality index	1.14	0.169–2.119	0.497
Written s*hura-e-sehie* activities	13.32	10.62–16.00	1.371
Health worker satisfaction index	2.26	1.32–3.21	0.481
Equipment functionality index	6.13	5.13–7.12	0.508
Drug availability index	3.67	2.03–5.3	0.832
Family planning availability index	9.04	7.36–10.72	0.858
Laboratory functionality index (hospital/CHC)	10.81	9.27–12.37	0.709
Staffing index	7.95	6.09–9.83	0.953
Provider knowledge score	5.00	4.11–5.88	0.452
Training in the past year	5.24	3.33–7.15	0.974
HMIS use index	7.27	5.37–9.17	0.971
Clinical guidelines index	12.43	10.97–13.89	0.743
Infrastructure index	1.25	0.08–2.4	0.593
Patient record index	1.72	0.05–3.39	0.851
Facilities with TB registers	13.25	10.89–15.61	1.203
Patient history and physical exam index	3.30	1.93–4.69	0.705
Patient counseling index	3.42	1.64–5.21	0.909
Proper sharps disposal	5.69	2.58–8.79	1.583
Average new outpatient visit/month (BHC>750 visits)	10.84	7.69–13.99	1.606
Patient consultation time (≥10 min)	3.00	0.81–5.19	1.118
BPHS facilities providing antenatal care	9.87	7.63–12.12	1.144
Delivery care according to BPHS	12.58	10.51–14.65	1.056
Facilities with exemptions for poor patients^a^	5.62	3.47–7.78	1.100
Females as percent of new outpatients	1.67	1.24–2.1	0.219

All estimates *p<*0.001, except for patient perception of quality (*p<*0.02), infrastructure index (*p<*0.04), and patient record index (*p<*0.03).

a 2004–2007.

CHC, comprehensive health center.

### Staff Perspectives and Satisfaction Domain

Provider satisfaction included multiple measures of various health system attributes and support factors including training, professional development opportunities, availability of essential equipment and medicines according to BPHS guidelines, and working relationships with various stakeholders. There was a slight, but significant, increase in this index indicator (+6%) to about 70% in 2008. In 2008, median scores for providers reporting timely salary payments increased by 20% from baseline levels. Financial incentives and regular payments are important contributory factors for provider motivation and retention, and the finding suggests improved management of implementing agencies.

### Capacity for Service Provision

Compared with other domains, the most dramatic improvements were evident in the capacity for service provision domain (47.4–76.4, *p<*0.0001). All system preparedness factors for service provision; essential medicines, equipment, clinical guidelines, laboratory functionality, health provider adequacy, skill mix, and provider knowledge and training showed significant improvements since 2004. The evidence illustrates the investment efforts to adhere to BPHS standards to ensure capacity for service provision, a critical prerequisite for ensuring optimal quality of care and health outcomes. The annual progressive increase estimated by GEE was significant for all indicators in this domain (Table 3).

### Service Provision

Significant improvements were evident for adherence to clinical standards for patient history and physical exam as well as counseling, although the latter was still at suboptimal levels in 2008 (48% national median score). There was a progressive increase in average new patient visits per month, which reached peak levels (median score of 85) in 2008. Scores were significantly higher for provision of both antenatal (+36%, *p<*0.0001) and delivery care services (+46%, *p<*0.0001) from baseline levels. Despite these improvements, average patient consultation time diminished over the years, which may have resulted from higher levels of service utilization following the removal of user fees. Regression modeling demonstrated a strong statistical association between the capacity for service provision (coefficient 0.45, *p<*0.0001), with quality of service provision in 2008. Applying the GEE modeling, there was a significant increase (*p<*0.0001) in adherence to standards for patient history taking and patient counseling during the 5-y period.

### Financial Systems

Modest improvements were evident in the existence of patient user fee guidelines (+6.5% by 2007). All sampled facilities provided user fee exemptions for poor patients, raising the national median score to 100 in 2007, a 17.5% (*p<*0.0001) increase from baseline levels. Discussions are underway to reconfigure this domain to include other characteristics of health facility and hospital financial management.

### Overall Vision for Pro-poor and Pro-female Services

One of the primary goals of the BPHS was to ensure equity of service delivery to the poor and optimize utilization of females for outpatient services. A concentration index of 50 would indicate no differences in service utilization, and a higher index would indicate higher utilization of females or poorer patients. This objective was partially achieved, as female patients increasingly constituted a higher proportion of new outpatient visits (+4.9%, *p<*0.0001), but there was minimal improvement in scores for equity of utilization or patient satisfaction as measured through concentration indices of users over the years.

## Discussion

Despite the inherent health environment challenges and worsening security, the government of Afghanistan has made impressive gains in strengthening the capacity of the health system to improve service delivery, as evidenced by the increasing trends in various domains. Since its inception in 2004, the scorecard has been adopted by the MOPH, as a key performance management tool, demonstrating effective stewardship, to illustrate evidence of investments, and facilitate policy changes. Furthermore, it has created a learning organization, enhancing a more evidence-based decision-making culture [18],[20]. The following sections provide some evidence on the management utility and limitations of the BSC to manage health service delivery and some recommendations for optimizing the tool as a comprehensive health system performance measure.

### Leadership and Transparency

Lack of leadership support has often been cited as a barrier to successful scorecard implementation [10]. However, in the Afghanistan context, the support of the executive leadership in policy and planning was a key enabling factor for the significant achievements in national performance scores. The BSC served as a strategic management tool to ensure capacity, identify factors contributing to performance deficiencies, and determine policy and resource innovations to promote good governance [15]. Despite some initial skepticism and reluctance to adopt the tool, over the years there have been several “champions” who leveraged the interest and support of other investors in the health sector to sustain the application of the BSC. In a postconflict environment, heavily reliant on external donor assistance, the BSC has been instrumental in enhancing transparency in the decision-making process, creating a culture of accountability by policy leaders to manage performance-based contracts. Improved performance trends helped demonstrate positive results to the public to rebuild trust in the government health care system [13],[18].

### Evidence of Improved Health Service Capacity and Delivery

As a performance assessment tool, the BSC has provided a comprehensive framework of multiple service delivery and system elements, illustrating impressive improvements to achieve the BPHS standards for optimal service provision. The trends provide the evidence of investments to enhance health infrastructure, staffing and resource capacity, and subsequent impact on service provision. The customer domains of patient and provider satisfaction, and the financial domain (through 2007) also showed significant improvements during the 5 y. However, efforts must be made to motivate providers to improve counseling quality by providing appropriate instructions to patients on home care, conditions for immediate return to facility, and correct administration of prescriptions. Time spent on patient consultation and counseling, a significant predictor of quality of care [18],[21], showed a decreasing trend over the years with levels returning to baseline scores in 2008. Without additional health system level resource investments complemented by patient triaging, improving counseling quality will continue to be a challenge.

We believe that the apparent dramatic improvements for some indicators and minimal changes in others over the 5-y period have three plausible reasons: (1) The median performance score for service capacity and service provision domains was at much lower levels in 2004, (47 for service capacity and 40 for service provision) than the scores for overall patient satisfaction (>80 in 2004), patient perception of quality (>75% in 2004). Apparently the improvements were more dramatic for domains that had low scores in 2004, than those that were at higher levels. (2) The presentation of the scorecard illustrating poorly performing domains and the ongoing efforts of donor priorities to establish health infrastructure and resource capacity resulted in additional performance improvement strategies to improve the scores for these indicators. (3) The possibility of a courtesy bias resulting in high levels of patient satisfaction over the years has also been hypothesized in a later section.

### BSC Facilitated Performance Benchmarking

In addition to enhancing improvements in service capacity and delivery, the BSC facilitated historical and provincial benchmarking and determining the effectiveness of various contracting mechanisms [20]. Benchmarking performance has been demonstrated to be an effective strategy, to foster healthy competition and create a culture of evidence to determine factors of success or failure, particularly in decentralized health systems, and is still considered a relevant strategy for the Afghan context, despite the provincial variations in the health care environment particularly security and geographic access [22],[23].

### Remaining Challenges

Despite the progress on health system performance, Afghanistan continues to face challenges to ensure equitable service delivery. Service utilization for female patients is still dependent on the availability of female providers. Though the acceleration of the midwifery training programs has tried to meet the increasing demand for female providers, the data from the national assessments does not provide information on the national health workforce because of the sampling strategy employed. The human resource database indicated that female providers currently constitute only 24% of the workforce, with considerable variation across provinces. The overall proportion of health provider shortage estimated at 39% (based on BPHS standards) is still a severe deficit, compounded by worsening security [1]. Poor provider motivation can lead to suboptimal quality due to nonadherence to clinical guidelines, harmful practices, and poor provider–patient interactions [24]. Results-based financing mechanisms, similar to those implemented in Rwanda [25], have been introduced recently to incentivize providers to enhance motivation and performance. Though external assistance and public health spending has increased substantially in the last few years, better targeting of services is critical to ensure equity.

### Limitations in the Design and Execution of the BSC

It is also important to highlight some of the limitations in scorecard design and execution for policy makers to debate the utility of the Afghanistan scorecard tool to measure health service delivery. Aside from the classic Hawthorne bias of performance improvements due to the fact that providers are aware of being observed, the quality of care assessments did not have gold standards by which the accuracy of diagnosis or treatment could be ascertained. High levels of patient satisfaction in this context may also be attributed to a courtesy bias, as patients with limited physical and financial access to services, may fear the consequences of negative feedback, resulting in termination of services [26]. Leveraging the support of village health councils to ensure community feedback may alleviate some of this fear and enhance community trust of the health system.

Another limitation to the scorecard design is the lack of measures to assess the impact of investments at the population level, as currently the assessments are organized around health facilities and the users of services. Although the BSC was not designed to measure important health impact like mortality rates or to evaluate the causal relationships that link to such measures of population health, future strategies will try to link household surveys to the facility-based assessments to examine causal associations between facility interventions and observed changes in population-based outcomes. These could include effectiveness and efficiency measures to demonstrate the overall performance of the health system.

To ensure equitable access to services, the establishment of subcenters, mobile clinics, and health posts has accelerated in recent years with more than 22,000 community health workers trained and deployed nationally to provide health promotion and first level of care in rural communities. Efforts are underway to reconfigure the scorecards to integrate community provider performance measures and assess investments made to the base of the health system pyramid to demonstrate results as communities and patients engage to become coproducers of health [27],[28].

A commonly cited barrier of scorecard implementation is the progression from a measurement tool to a performance management tool for continuous quality improvement to ensure social accountability and balance other financial considerations [29]. It is hypothesized that these quality improvement strategies may offset some of the costs of BSC reconfigurations necessitated by the changing health care environment [9]. The national quality assurance committee in Afghanistan, has been instrumental, in prioritizing performance deficiencies identified from routinely obtained health management information and supervision systems, as well as the NHSPA, in order to identify and address management constraints [30]. We recognize the importance of rigorous survey designs to assign cause and effect to improvements instituted as it provides a record of strategic inflection points when policies changes and resources were appropriated to improve systems and performance. Deficient performance such as inadequate provider knowledge or stock out resulted in improved efforts for refresher training and ensuring adequate stocks of essential drugs and review of NGO performance when issuing new contracts [18].

### Cascading Scorecard Results to Frontline Providers

Translating the scorecard strategy into easily comprehensible performance measures to demystify the scorecard at the peripheral levels is important for the frontline providers to comprehend the value of the measurement strategy [11]. In Afghanistan, dissemination of scorecard results was primarily targeted to the policy leaders and contracting and implementing agencies responsible for the oversight of the health system at the national and provincial level, but there was minimal evidence on its use at the facility level. The importance of packaging and cascading the scorecard results to the frontline constituents cannot be undermined as this is critical for creating autonomy in the peripheral units to address context specific performance deficiencies through rapid quality improvement cycle efforts [9],[11].

### Impediments in the Health Care Environment

The lack of explicit systems and policies, minimal infrastructure, high corruption levels, low literacy rates, and resistance to measurement, identified as barriers for successful scorecard execution, are also inherent in Afghanistan's health care environment [31]. However, the major challenge will be to sustain the momentum of BSC performance assessments in the context of worsening security, and therefore the long-term effectiveness of the current scorecard system warrants further scrutiny, particularly if donor assistance diminishes in the future. Further analysis applying multivariate statistical modeling controlling for confounders will be required to determine the major contributors for improving health service delivery in this context [32].

### Future Scorecard Reconfigurations

Transitions in the health system landscape of leadership and political priorities, changes in the health system architecture of financing and resource structures, guidelines and policies, epidemiological transitions, and security constraints will necessitate periodic reconfigurations to ensure the scorecard's relevance and effectiveness. The Ministry and its stakeholders are considering the integration of indicators to determine effectiveness of financial management operations and performance to replace the user fee indicators formerly in the financial management domain. As all provinces have achieved the 2004 performance benchmarks, new targets will be required in the future, as well as the inclusion of indicators for emerging health priorities to modify the scorecard.

The costs for conducting the annual national performance assessment to generate the scorecards are estimated at 2.5% of the overall cost of delivering the BPHS. As the government becomes more self-reliant, the scorecard may be scaled down to a few key performance indicators that can be derived from data obtained from health information systems supplemented by routine rapid facility assessments to minimize survey costs.

### Enhanced Organizational Learning Promoting a Culture of Measurement

Few national performance measures were available when the BSC was first initiated for the primary health care sector. As the health system matured, several parallel measurement systems were standardized using the BSC prototype, including scorecards to determine performance of the essential package of hospital services and more recently the ongoing routine assessments for the health management information systems. The scorecard enables a single organizational measure, and reduces the burden of managing fragmented performance assessments focused on few health service delivery elements. All surveys and measures have been conducted at the national level to include all secure provinces addressing vertical scale-up and assessment mechanisms for inclusion of insecure provinces are in consideration.

### Conclusion

Emerging from decades of war and continued insecurity, Afghanistan has successfully pioneered the integration of the BSC at the national and provincial levels, to improve the delivery of basic health services. The BSC provides a dashboard of indicators integrating the building blocks of health systems, promoting effective governance and leadership, facilitating good stewardship and managerial autonomy for timely decisions on policy and service delivery [33]. Despite the promising results so far, the successful execution of the BSC will depend on its adaptive ability and sustained efforts of the MOPH leadership to accommodate dynamic and complex changes in the health care environment. As the global momentum for health systems strengthening accelerates, the balanced scorecard offers a promising measure of comprehensive health system performance to examine the effectiveness of strategies and innovations executed, particularly in resource constrained environments.

## Abbreviations

BPHS: Basic Package of Health Services
BSC: balanced scorecard
GEE: generalized estimating equation
MOPH: Ministry of Public Health
NGO: nongovernmental organization
NHSPA: National Health Service Performance Assessment
